# Nasal Outcomes of Presurgical Nasal Molding in Complete Unilateral Cleft Lip and Palate

**DOI:** 10.1155/2012/643896

**Published:** 2012-09-11

**Authors:** Emily M. Williams, Carla A. Evans, David J. Reisberg, Ellen A. BeGole

**Affiliations:** ^1^Department of Orthodontics, University of Illinois at Chicago, Chicago, IL 60612, USA; ^2^The Craniofacial Center, The University of Illinois Medical Center at Chicago, Chicago, IL 60612, USA

## Abstract

*Objective*. Short-term nasal forms following primary lip repair were compared between presurgical nasal molding and control groups. *Aim*. To compare nasal symmetry between patients that had nasal molding and lip repair with those that had only lip repair. *Design*. Retrospective case-control study Patients. Complete unilateral CL+P patients had basilar and frontal photographs at two time points: (1) initial (2) postsurgical. 28 nasal molding patients and 14 control patients were included. Intervention. Presurgical nasal molding was performed prior to primary lip repair in intervention group. No nasal molding was performed in control group. *Hypothesis*. Nasal molding combined with lip surgery repair according to the Millard procedure provides superior nasal symmetry than surgery alone for nostril height-width ratios and alar groove ratios. *Statistics*. Shapiro-Wilk test of normality and Student's *t*-tests. *Results*. A statistically significant difference was found for postsurgical nostril height-width ratio (*P* < .05). No other statistically significant differences were found. *Conclusions*. Nasal molding and surgery resulted in more symmetrical nostril height-width ratios than surgery alone. Alar groove ratios were not statistically significantly different between groups perhaps because application of nasal molding was not early enough; postsurgical nasal splints were not utilized; overcorrection was not performed for nasal molding.

## 1. Introduction

Use of presurgical nasoalveolar molding (NAM) and similar orthopedics in the management of cleft deformities has been a subject of occasional controversy [1, 2]. Appliance effectiveness, cost, and treatment time have been previous subjects of debate. Mastuo and Hirose [3] recognized the moldability of nasal cartilages in the early months of an infant's life and attributed this to high levels of estrogen and increased hyaluronic acid. They are credited with the first attempt to perform nasal molding on patients with cleft lip and palate by using silicone rubber stents in the nostrils. Within a few years, Grayson et al., [4] developed an appliance with a nasal extension attached to the anterior portion of an acrylic alveolar molding plate, which marked the advent of presurgical nasoalveolar molding appliances. A modification of the Grayson-type NAM appliance is utilized by The Craniofacial Center at the University of Illinois Medical Center. An acrylic bulb at the end of a wire is embedded in the plate and can be adjusted in conjunction with alveolar molding. The appliance is intraorally retained by denture adhesive on the palatal surface, and extraoral tapes are not utilized. The University of Illinois cites several advantages in their design including ease of fabrication and adjustment by the operator, less airway obstruction with the smaller acrylic extension, and parental preference for the less noticeable appearance of the appliance design [5]. The University of Illinois uses the NAM appliance solely for nasal molding. Assessing outcomes of presurgical appliances may help determine their value.

Very recently, the only reported systematic review on presurgical infant orthopedics (PSIO) was released concerning long-term advantages of these appliances. The authors concluded that until the age of 6, there were no positive effects on factors such as facial growth, maxillary arch dimension, or occlusion when treatment included passive infant orthopedic appliances. However, the authors made the distinction between PSIO and NAM appliances, stating that their review yielded the conclusion that nasal symmetry was improved with NAM and that more randomized clinical trials should be conducted to assess long-term nasal symmetry outcomes [6].

When describing attractive faces, the literature stresses the importance of facial symmetry [7–9]. Facial symmetry increases attractiveness [10]. The visual impact of symmetry has been shown to be more critical toward the midline [11], which is unfortunate for individuals with cleft lip and palate since their greatest deformities are near the midline of the midface; these asymmetries have been shown to produce more negative evaluation of the facial esthetics [12]. Several studies have demonstrated improved nasal symmetry following presurgical NAM [13–19]. Although the University of Illinois has been performing nasal molding with the NAM appliance since the 1990s, no results were reported by this institution and the quantitative assessment of outcomes for nasal symmetry in this study is therefore valuable and relevant to what has been previously documented as being esthetically important. 

## 2. Materials and Methods

The Institutional Review Board at the University of Illinois at Chicago approved the protocol for this study. Records of patients were obtained from the Craniofacial Center at the University of IL,USA, Medical Center, Chicago, Illinois and from a private orthodontic practice in Miami, FL, USA. Subjects must have undergone primary lip repair within the past 70 years. Syndromic patients were excluded. Infant and children patients aged 0–3 years old who have complete unilateral cleft lip and palate and have presurgical and postsurgical frontal and basilar photographic records were included. Presurgical records were taken on initial evaluation of each patient, prior to initiation of any molding treatment or procedure. Postsurgical records must have been obtained within two years of the primary lip repair. A total of forty-two nonsyndromic patients with complete unilateral cleft lip and palate were selected for this study. Twenty-eight patients underwent presurgical nasal molding without taping prior to primary lip repair, while fourteen patients did not undergo any presurgical orthopedics and only had primary lip repair. Of the fourteen control patients, seven were patients of the University of Illinois and the remaining seven were patients of the private orthodontic practice. The same surgeon performed lip repair for all patients in the nasal molding group. This same surgeon also performed lip repair for a portion of the control subjects from the University of Illinois, while a second surgeon performed lip repair on the remaining controls from the University of Illinois. A third surgeon performed lip repair for all seven control subjects from the private orthodontic practice. All surgeons performed lip repair according to the Millard procedure. The mean ages for presurgical records were 2.8 weeks for the nasal molding group and 2.6 weeks for the control group, and mean ages for postsurgical records were 6.5 months for the nasal molding group and 6.9 months for the control group.

**Figure 1:**
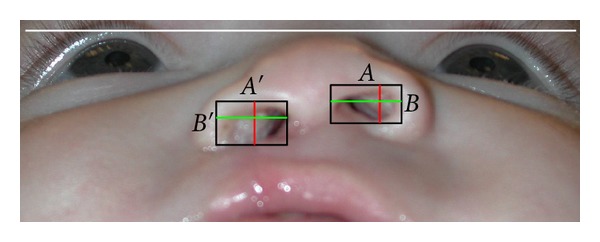
Nostril height and width ratio = the nostril height and width ratio on the cleft side (*A*′/*B*′)/the nostril height and width ratio on the noncleft side (*A*/*B*). For clarification of Figure 1, in this study *A*′ was cleft side width, *B*′ was cleft side height, *A* was noncleft side width, and *B* was noncleft side height.

**Figure 2:**
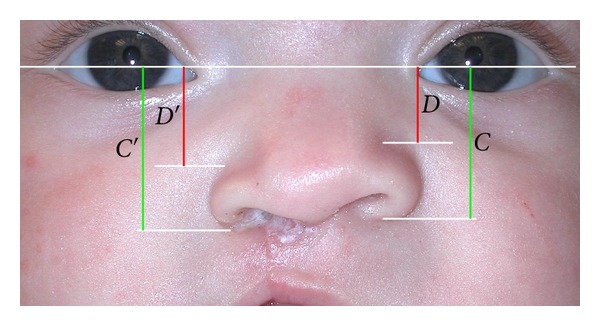
The distances between the nasal base and top of alar groove to the line intersecting medial ocular angles between sides were assessed. The ratio of the height of the alar groove = the ratio of the height of the top of the alar groove on the cleft side (*D*′/*C*′)/the ratio of the height of the top of the alar groove on the noncleft side (*D*/*C*).

Within the total 42 subjects, there were 17 females and 25 males; 18 were Caucasian, 12 were Black, 11 were Hispanic, and one was Asian; 22 were left-sided clefts, while 20 were right-sided clefts. Within the nasal molding subjects, there were 11 females and 17 males; 7 were Caucasian, 11 were Black, 9 were Hispanic and 1 was Asian. Within the control subjects, there were 6 females and 8 males; 11 were Caucasian, 1 was Black, 2 were Hispanic, and none were Asian. Photographs of infants' noses in basilar and frontal views were collected from both pre- and postsurgical time points. All images were digitally scanned, cropped to include only partial facial images, and printed in color on white paper. Removal of the identification of the records according to the Health Insurance Portability and Accountability Act ensured that no patient was identified by the principal investigator. Nasal forms were assessed by means of direct measurement with a digital caliper on each printed photograph for nostril height-width ratios and alar groove height ratios, between cleft and noncleft sides, at each time point based on formulas as seen in Figures 1 and 2. All measurements were repeated by the author on a separate day and were all within 0.3 mm of the first measurements.

The methods for measurements of both nostril height-width ratios and alar groove height ratios were similar to the study performed by Nakamura et al. [16]. Utilizing ratios for each measurement minimized inconsistencies with photographic archives which may have been due to calibration or magnification errors between subjects or within the time points of a particular subject. For statistical analysis, a Shapiro-Wilk test was performed and all ratio measurements at each time point were compared with Student's *t*-tests (Table 1). Significance was accepted at *P* < .05.

## 3. Results

Shapiro-Wilk test confirmed normality for presurgical and postsurgical data at *P* < .05.

### 3.1. Nostril Height-Width Ratios

For nostril height-width ratios, no statistically significant difference was found in presurgical ratios (PCNWR) between nasal molding and control groups. Groups were initially similar with regard to severity of clefts based on statistical analysis. For postsurgical ratios (OCNWR), a statistically significant difference was found between nasal molding and control groups. The mean OCNWR was 1.46 for the nasal molding group and 1.91 for the control group. A 1 : 1 ratio, or 1.0 numerical mean, would represent the highest achievable symmetry between sides. Since the nasal molding group mean score was closer to 1.0 than the control group, it can be assumed that more symmetrical outcomes for the nasal molding group were obtained with regard to nostril height-width ratios.

### 3.2. Alar Groove Height Ratios

For alar groove height ratios, no statistically significant difference was found in either presurgical ratios (PCNAR) or postsurgical ratios (OCNAR) between nasal molding and control groups. Initially groups were similar with regard to severity of clefts based on statistical analysis. The mean OCNAR was 1.10 for the nasal molding group and 1.14 for the control group. There was no statistically significant difference between groups in the present study. 

## 4. Discussion

In this study, nostril height-width ratios were calculated by the formula (*A*′/*B*′)/(*A*/*B*), where “*A*” measurements were width and “*B*” measurements are heights. Nakamura et al. [16] used the same formula to calculate ratios, however “*A*” measurements were height and “*B*” measurements were width. The nostril height-width ratios can still be compared between studies by simply taking the inverse ratios for either one of the studies. When inverting the OCNWR measurements for the present study, 1.46 becomes 0.68 for the nasal molding group and 1.91 becomes 0.52 for the control group. Nakamura et al. [16] reported postoperative ratios of 0.76 for the nasal molding group and 0.61 for their control group, which was statistically significant at *P* < .01. The results from both studies represent superior outcomes for nasal molding groups compared to control groups for nostril height-width ratios, however, Nakamura et al. [16] reported ratios closer to 1 : 1 than the results of the present study, which could be interpreted to mean that their outcomes were more symmetrical than the present study.

In the alar groove height ratios, mean OCNAR was 1.10 for the nasal molding group and 1.14 for the control group, which was not statistically significant different. Nakamura et al. [16] reported one-year postoperative alar groove height ratios of 1.03 for NAM and 1.13 for controls, but the difference in their results was statistically significant. One must contemplate possible reasons for the lack of statistically significant difference between our nasal molding and control groups for alar groove height ratios. Bennun et al. [20] suggested that very early application of NAM by the first two days of life resulted in more symmetrical long-term nasal outcomes than initial NAM application beyond two weeks of age. In our study, the absence of adhesive tape used in conjunction with nasal molding may have resulted in inadequate alar suspension on the cleft side in our population. Use of postsurgical nasal splint appliances for at least six months postoperatively have been advocated by Yeow et al. [21] and Chang et al. [22] to prevent relapse following NAM. These nasal splints help maintain the alar cartilage height and prevent collapse during scar healing and beyond. Wakami et al. [23] proposed the application of a presurgical nostril suspension device consisting of extraoral tape affixed to the forehead of the infant connected to paper clips which lift the alar cartilage. The infants also wore nasal retainers for six months postoperatively, and the authors reported improved ratings for both nostril symmetry and alar cartilage position in the infants treated with their suspension device. The nasal molding subjects in the present study had a mean age at initial records of 2.82 weeks, which may have been later than ideal to start molding per Bennun et al. [20]; however, Shetty et al. [24] advocate positive effects of presurgical NAM can still be achieved when initiated between one and five months of age. Additionally, without postoperative nasal retention, the subjects may have shown tendency toward relapse. Subjects in this study may have also benefited from a nostril suspension device as described by Wakami et al. [23]. Another plausible reason that alar groove ratios were not statistically significantly different in our study was that overcorrection was not performed on nasal molding subjects. Chang et al. [22] suggested that overcorrection of 20% maintained nostril height after 5 years, but that NAM alone could not provide nostril symmetry in the long-term. 

The University of Illinois does not turn away potential nasal molding subjects based on ability of parents to pay for treatment. UIC also serves a large Medicaid population that may not receive treatment from other institutions that have a Medicaid “quota” or refuse such patients. Subjects of this study may have perhaps been denied care if they lived in a different region, and these results may have gone undocumented. By providing care for such underserved demographic groups, bias and lack of reporting is potentially reduced compared to other centers. Sischo et al. [25] suggested that cleft services may be linked to ethnicity in that African American and Latinos from their study demonstrated tendency toward selecting traditional, non-NAM care when offered a choice. The present study as well as future studies from our institution could represent a population with more racial and/or socioeconomic diversity. Additionally, the University of Illinois does not prescribe to primary bone grafting or gingivoperiosteoplasty in its surgical protocol, and the results from this study should be used to compare to other centers that do utilize such surgical procedures. Even without surgical supplement beyond primary lip repair, nostril height-width ratios were superior in the nasal molding group in this study.

There were obvious limitations to this study, most of which stem from the retrospective nature of this study. Since outcomes from three different surgeons were assessed, the variations in surgical technique or operator skill contribute possible uneven distribution within groups and results. In the present study, all lip repairs were performed according to the Millard procedure, which the authors feel reduces variation based on surgical type. Ideally, one surgeon would have performed all surgeries for control and NAM subjects, but this factor could not be controlled. Additionally, data was collected from a span of the past 70 years. It would have perhaps been optimal to have all records taken within a more recent time frame, but due to the difficulty in finding adequate quantities of control subjects this was not possible. Also, while it would be valuable to assess nasal changes over a longer period of time following surgical repair, only short-term records were available to the author. Observing changes due to growth and maturation would be important aspects of a future study if follow-up records became available. Only two-dimensional photographs were available in this study and were typical records taken for documentation of patients' progress in the past. More recently, 3D imaging has been utilized at the institution, and future studies may benefit from analysis of these data [26].

## 5. Conclusions

In this study, nasal molding subjects had superior postsurgical nostril symmetry compared to controls in relation to nostril height : width ratios. Alar groove height symmetry, on the other hand, was not found to be different between nasal molding and control subjects. The lack of difference for alar groove height symmetry may be due to a delay beyond two weeks of life for initiation of nasal molding activation, lack of nasal splints for retention, or failure to overcorrect alar cartilage molding prior to surgery in order to prevent relapse from occurring. 

This study investigated only short-term nasal symmetry outcomes after presurgical nasal molding. Long-term assessment of nasal molding is necessary to determine its effects on facial and nasal growth as well as patient self-perception of nasal esthetics. Additionally, long-term studies are needed to analyze whether nasal molding truly reduces the need for future nasal revision or other health care costs with age. As the field of cleft lip and palate care evolves, many NAM opponents may continue to argue its efficacy unless consistently positive results emerge from the literature. This study demonstrated that, for the short-term, nasal molding with a NAM-type appliance was effective in providing symmetrical nostril outcomes. Support for continuation of NAM and for the future improvement of its protocol is therefore warranted.

## Figures and Tables

**Table 1:** Comparison of ratio means between groups.

Measurement	Nasal Molding		Control			
	Mean	St Dev	Mean	St Dev	*t*	*P*
PCNWR*	4.90	3.607	4.20	1.956	−0.679	0.501
OCNWR	1.46	0.466	1.91	0.809	2.330	0.025
PCNAR	1.44	1.93	1.37	0.203	−1.102	0.277
OCNAR	1.10	0.103	1.14	0.145	1.084	0.285

*PCNWR: presurgical cleft nostril height-width ratio; OCNWR: postsurgical cleft nostril height-width ratio; PCNAR: presurgical cleft nose alar groove height ratio; OCNAR: postsurgical cleft nose alar groove height ratio.
